# Multi-Observer Fusion Based Minimal-Sensor Adaptive Control for Ship Dynamic Positioning Systems

**DOI:** 10.3390/s25030679

**Published:** 2025-01-23

**Authors:** Yanbin Wu, Xiaomeng He, Linlong Shi, Shengli Dong

**Affiliations:** 1 College of Information Science and Technology, Dalian Maritime University, Dalian 116026, China; wuyanbin@dlmu.edu.cn; 2 College of Marine Electrical Engineering, Dalian Maritime University, Dalian 116026, China; 3 Shanghai KeLiang InformationTechnology, Co., Shanghai 200233, China; linlong.shi@keliang.com; 4 Shanghai Ship and Shipping Research Institute Co., Shanghai 200135, China; dong.shengli@coscoshipping.com

**Keywords:** multi-observer, minimal-sensor control, adaptive dynamic positioning, neural network learning, SimuNPS

## Abstract

This paper proposes an adaptive dynamic positioning (DP) control method based on a multi-observer fusion architecture with minimal sensor requirements. A sliding mode observer is designed based on a high- and low-frequency superposition model to filter high-frequency state variables, while a finite-time convergence disturbance observer estimates unknown time-varying low-frequency disturbances online. For efficient handling of model uncertainties, a single-parameter learning neural network is implemented that requires only one parameter to be estimated online. The control system employs auxiliary dynamic systems to handle input saturation constraints and considers thruster system dynamics. Theoretical analysis demonstrates the stability of the observer-fusion control strategy, while simulation results based on the SimuNPS platform validate its effectiveness in state estimation and disturbance rejection compared to traditional sensor-dependent methods.

## 1. Introduction

Dynamic positioning (DP) systems enable vessels to autonomously maintain their position and heading through onboard thrusters and lateral propulsion systems to counteract environmental forces such as wind, waves, and currents. With depth-independent operation, high positioning accuracy, and strong maneuverability, DP systems are extensively employed in unmanned surface vessels, supply ships, rescue vessels, and oil and gas drilling platforms, making them indispensable for deep-sea resource exploration and overcoming the limitations of traditional anchor-based positioning in deep-sea operations [1,2]. Traditionally, these DP systems heavily rely on various sensors for state measurement and environmental monitoring. However, the installation and maintenance of multiple sensors increase system complexity and cost, while sensor failures may compromise system reliability [3]. Therefore, developing control strategies with minimal sensor requirements for DP systems has become an important research direction in marine engineering.

In complex oceanic environments, DP vessels encounter various disturbances. Unknown, time-varying low-frequency disturbances from second-order wave forces generated by wind, currents, and waves affect the vessel’s low-frequency motion [4], while first-order wave forces lead to high-frequency motion through wave-induced disturbances [5]. Additionally, model uncertainties and parameter variations within the DP system introduce further disturbance factors [6]. Each type of disturbance affects DP vessels through distinct mechanisms, resulting in varied impacts. However, traditional DP systems face several key limitations in addressing these challenges. First, the heavy reliance on multiple sensors increases system complexity, maintenance costs, and vulnerability to sensor failures [3]. Second, conventional Kalman filter-based approaches for high-frequency motion filtering show reduced robustness when confronted with sea state variations and vessel parameter perturbations. Third, existing neural network implementations for uncertainty handling often impose a significant computational burden, potentially compromising real-time performance. Thus, anti-disturbance strategies must be carefully designed to address the unique characteristics of each disturbance type while overcoming these limitations. Consequently, focused research on minimal-sensor control strategies to mitigate first-order wave-induced disturbances, unknown time-varying low-frequency disturbances, and additional system disturbances is essential for achieving robust DP.

Recent advances in observer design and minimal-sensor control have demonstrated promising results in various marine applications. Particularly, observer-based approaches have shown potential in achieving high-precision state estimation with minimal sensor requirements. In conventional DP systems, the predominant approach to vessel high-frequency motion filtering relies on Kalman filters for state estimation. However, these filters exhibit reduced robustness when confronted with sea state variations and vessel parameter perturbations, limiting their effectiveness in managing uncertainties. Research has demonstrated that sliding mode observers (SMOs) offer superior capabilities in handling such uncertainties and external disturbances, particularly in high-frequency disturbance suppression [7]. SMOs achieve more precise state estimation and demonstrate enhanced robustness compared to traditional Kalman filters, featuring finite-time disturbance convergence characteristics. The challenge of unknown time-varying low-frequency disturbances has been addressed through nonlinear disturbance observer (NDO) based control methodologies, which significantly enhance system robustness. Notable applications include the integration of NDO technology with backstepping approaches in quadrotor UAV control systems [8], where these observers can effectively estimate high-frequency disturbances, with estimation errors converging within a bounded spherical region near equilibrium points [9]. Recent studies have demonstrated the effectiveness of multi-observer approaches in various control applications. Li and Wei [10] developed a multi-observer framework with neural networks that achieved robust state estimation under sensor attacks. For marine applications, Gu et al. [11] comprehensively reviewed disturbance observer techniques, highlighting the importance of integrated observer design. Additionally, Zheng et al. [12] proposed an adaptive fusion framework for multiple ESOs that effectively handles estimation peaks and measurement noise. Meanwhile, in the field of control theory, significant advances have been made in finite-time control and its applications [13], including event-triggered adaptive control [14], command filter-based control [15], and finite-time tracking control with unmodeled dynamics [16]. Inspired by these developments in both observer design and finite-time control, this paper proposes a multi-observer fusion framework for marine vessel dynamic positioning that combines the advantages of both approaches.

To combat additional disturbances stemming from vessel model parameter perturbations, neural network approaches have emerged as effective solutions [17,18]. Traditional implementations have utilized radial basis function neural networks (RBFNN) and fuzzy neural networks to approximate compound uncertainties. Specific applications include RBFNN-based controllers for yaw angle and speed tracking [19] and disturbance suppression systems [20]. However, these conventional neural network implementations often impose significant computational demands, compromising system complexity and real-time performance. Recent developments have introduced more efficient neural network architectures to address these computational challenges. Adaptive algorithms based on multilayer perceptrons (MLP) have reduced parameter estimation requirements to just 2–3 weight parameters [21,22,23,24,25,26]. A particularly noteworthy advancement is the single-parameter learning neural network [27], which achieves compound uncertainty linearization through inequality transformations. This innovative approach, requiring only one parameter estimation, substantially reduces computational overhead while maintaining system effectiveness.

Beyond environmental disturbances, vessel control systems face significant challenges from input saturation constraints, necessitating comprehensive investigation into thruster system characteristics and physical limitations. Research efforts have yielded diverse DP control methodologies to address these challenges [28,29,30]. A notable advancement includes the development of a specialized proportional-integral anti-saturation control framework designed specifically for DP applications [28]. Further research has successfully integrated anti-saturation techniques with integral actions, simultaneously addressing both saturation constraints and constant disturbance compensation [29]. In the realm of control system architecture, the implementation of auxiliary power systems has emerged as an effective approach for managing input saturation while enforcing thruster control signal constraints. Recent developments have focused on enhancing system responsiveness through finite-time auxiliary power systems, which guarantee auxiliary state convergence within defined time boundaries, representing a significant advancement in auxiliary system design [31]. A breakthrough in this field was achieved by Hu et al. [32], who pioneered an innovative approach incorporating thruster system dynamics directly into DP control frameworks, marking a substantial advancement in control system design.

Based on the above analysis, traditional DP systems face several fundamental challenges: the heavy reliance on multiple sensors leads to system complexity and reliability concerns; conventional observers show limited robustness in handling varied disturbances; and existing neural network methods impose significant computational burdens. To address these limitations, this paper proposes a minimal-sensor control framework based on multi-observer fusion for unmanned surface vessel DP. The main contributions are as follows:

1. A multi-observer fusion-based control framework is proposed, achieving high-precision state estimation and disturbance rejection with reduced sensor dependency. The system effectively processes high-frequency and low-frequency components through dual-observer architecture.
2. The state of the thruster is fully considered. The auxiliary dynamic system is utilized to address the input saturation problem, while the dynamic equations of the thruster system are established to characterize its dynamic behavior.
3. The comprehensive challenges that DP systems encounter in complex sea conditions are addressed through a single-parameter learning neural network for model uncertainties, requiring only one parameter to be estimated online.

The structure of the present study is as follows. In Section 2, the high and low frequency linear superposition model for DP ships and the basic knowledge required for controller design are presented. In Section 3, an adaptive nonlinear output feedback control law based on a sliding mode observer was designed; Section 4 demonstrates the effectiveness and stability of the control system. In Section 5, the effectiveness of the control system was verified through simulation; Section 6 provides a comprehensive discussion of the results; Section 7 is the conclusion.

## 2. Problem Formulation and Preliminaries

The dynamic positioning system with thruster dynamics can be expressed as [33]:

(1) $$\dot{\eta }=R\left(\psi \right)\upsilon ,$$

(2) $$M\dot{\upsilon }=-D\upsilon +\tau +d,$$

(3) $$\dot{\tau }=-A_{tr}\tau +A_{tr}\tau_{p},$$

where $\eta ={\left[x,y,\psi \right]}^{T}$ represents the vessel position and heading angle in the North-East-Down reference frame, with *x*, *y* denoting the position coordinates and ψ the heading angle. $\upsilon ={\left[u,v,r\right]}^{T}$ denotes the vessel velocity vector in the body-fixed frame, where *u*, *v* are the surge and sway velocities, and *r* is the yaw rate. The rotation matrix $R\left(\psi \right)\in R^{3\times 3}$ is given by:

(4) $$R\left(\psi \right)=\left[\begin{array}{ccc} \cos\psi & -\sin\psi & 0\\ \sin\psi & \cos\psi & 0\\ 0 & 0 & 1 \end{array}\right],$$

with properties $R^{-1}\left(\psi \right)=R^{T}\left(\psi \right)$ and $∥R(\psi )∥=1$.

The inertia matrix $M\in R^{3\times 3}$ includes both rigid-body and added mass terms, while $D\in R^{3\times 3}$ represents the hydrodynamic damping effects. $\tau ={\left[\tau_{1},\tau_{2},\tau_{3}\right]}^{T}$ denotes the control forces and moments generated by the thrusters. The vector *d* represents the combined environmental disturbances from waves, wind, and ocean currents.

(5) $$\begin{array}{cc} M= & \left[\begin{array}{ccc} m-X_{\dot{u}} & 0 & 0\\ 0 & m-Y_{\dot{v}} & mx_{c}-Y_{\dot{r}}\\ 0 & mx_{c}-N_{\dot{v}} & I_{z}-N_{\dot{r}} \end{array}\right],\\ D= & \left[\begin{array}{ccc} -X_{u} & 0 & 0\\ 0 & -Y_{v} & -Y_{r}\\ 0 & -N_{v} & -N_{r} \end{array}\right], \end{array}$$

where *m* is the vessel mass, $I_{z}$ is the moment of inertia about the z-axis, and $x_{c}$ is the x-coordinate of the center of gravity. The terms $X_{\dot{u}}$, $Y_{\dot{v}}$, etc. represent the hydrodynamic derivatives.

The matrix $A_{tr}\in R^{3\times 3}$ characterizes the thruster dynamics, and $\tau_{pi}$ represents the constrained control signals subject to actuator saturation:

(6) $$\begin{array}{c} \tau_{pi}=\mathrm{sat}\left(\tau_{ci}\right)=\left\{\begin{array}{cc} \mathrm{sgn}\left(\tau_{ci}\right)\tau_{Mi} & \left|\tau_{ci}\right|\geq \tau_{Mi}\\ \tau_{ci} & \left|\tau_{ci}\right|<\tau_{Mi} \end{array}\right.,i=1,2,3, \end{array}$$

where $\tau_{Mi}>0$ denotes the saturation limits for each thruster, $\tau_{c}={\left[\tau_{c1},\tau_{c2},\tau_{c3}\right]}^{T}$ is the commanded thrust vector, and $\Delta \tau =\tau_{p}-\tau_{c}$ represents the difference between the saturated and commanded thrusts. To account for model parameter uncertainties, we define $M=M_{0}+\Delta M$ and $D=D_{0}+\Delta D$, where $M_{0}$ and $D_{0}$ represent the nominal values, while ΔM and ΔD denote the parametric uncertainties. The DP system can then be reformulated as:

(7) $$\begin{array}{cc} \dot{\eta } & =R(\psi )v,\\ M_{0}\dot{v} & =-D_{0}v+\tau +d+\Delta ,\\ \dot{\tau } & =-A_{tr}\tau +A_{tr}\tau_{p}, \end{array}$$

where the lumped uncertainty term is defined as:

(8) $$\Delta =-\Delta M\dot{v}-\Delta Dv.$$

Given the finite energy nature of marine environments, the unknown time-varying environmental disturbances are assumed to be bounded:

(9) $$∥d∥\leq d^{*}<\infty ,$$

where $d^{*}$ is a positive constant.

To model the wave-induced motions, we introduce a high-frequency model:

(10) $$\dot{\xi }=A_{\omega }\xi +E_{\omega }\omega_{\omega },$$

(11) $$\eta_{w}=C_{\omega }\xi ,$$

(12) $$\dot{b}=-T_{b}^{-1}b+E_{b}\omega_{b},$$

where ξ represents the wave-induced states, $\eta_{w}$ denotes the wave-induced motion, and *b* characterizes the bias terms. The matrices $A_{\omega }$, $E_{\omega }$, $C_{\omega }$, $T_{b}$, and $E_{b}$ are of appropriate dimensions, and $\omega_{\omega }$, $\omega_{b}$ represent zero-mean Gaussian white noise processes.

The bias term can alternatively be modeled as a Wiener process:

(13) $$\dot{b}=\omega_{b}.$$

The measurement model combining both low- and high-frequency components is given by

(14) $$y=\eta +\eta_{w}=\eta +C_{\omega }\xi .$$

The assumption and lemma below are essential for the subsequent controller design:

**Remark** **1.** *For the DP system model (7), we make the following assumption: The model uncertainty terms ΔM and ΔD are bounded and represent structured uncertainties in the system matrices M and D.*

**Lemma** **1.** *Consider a positive definite continuous Lyapunov function* V(x,t) *defined on* $U_{1}\in R^{n}$. *If its time derivative satisfies* [34]:

(15) $$\dot{V}\left(x,t\right)\leq -c_{1}V^{\alpha }\left(x,t\right)-c_{2}V\left(x,t\right),\;\;\forall x\in U_{1},$$

*where $c_{1},c_{2}>0$ and 0<α<1, then the system achieves finite-time stability.*

## 3. DP Adaptive Output Feedback Control Design

**Figure 1:**
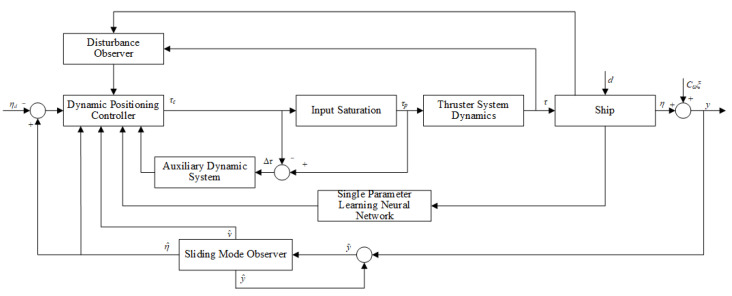
Block diagram of the proposed minimal-sensor adaptive DP control system.

This section presents the design of a control framework based on multi-observer fusion with minimal sensor requirements. The proposed approach achieves state estimation and disturbance rejection with reduced sensor dependency. The proposed control framework integrates a sliding mode observer, a disturbance observer, and a single-parameter learning neural network to achieve state estimation and disturbance rejection with reduced sensor dependency, as shown in Figure 1.

### 3.1. Design of Sliding Mode Observer

In this part, a sliding mode observer is proposed:

(16) $$\dot{\hat{\xi }}=A_{\omega }\xi +H_{1}\tilde{y},$$

(17) $$\dot{\hat{\eta }}=R(\psi )\hat{v}+H_{2}\tilde{y},$$

(18) $$\hat{b}=-T_{b}^{-1}\hat{b}+H_{3}\tilde{y},$$

(19) $$M\dot{\hat{v}}=-D\hat{v}+R^{T}(\psi )\hat{b}+\tau +R^{T}(\psi )H_{4}\tilde{y}-\kappa tanh\left(S_{0}\right),$$

(20) $$\hat{y}=\hat{\eta }+C_{\omega }\hat{\xi },$$

where $\tilde{y}=y-\hat{y}$ is the estimation error, the sliding mode surface of the sliding mode observer is defined as $S_{0}=v-\hat{v}$, $H_{1}\in R^{6\times 3}$, $H_{2},H_{3},H_{4}\in R^{3\times 3}$ is the observer coefficient matrix, and $\kappa =diag\left[\begin{array}{ccc} \kappa_{1} & \kappa_{2} & \kappa_{3} \end{array}\right]>0$ is the sliding mode surface gain of the observer.

Formulas (14), (15) and (18) can be written as

(21) $${\dot{\hat{\eta }}}_{0}=A_{0}{\hat{\eta }}_{0}+B_{0}R(\psi )\hat{v}+H\tilde{y},$$

(22) $$\hat{y}=C_{0}{\hat{\eta }}_{0},$$

where, ${\hat{\eta }}_{0}={\left[\begin{array}{cc} {\hat{\xi }}^{T} & {\hat{\eta }}^{T} \end{array}\right]}^{T}$, $A_{0}=\left[\begin{array}{cc} A_{\omega } & 0\\ 0 & 0 \end{array}\right]$, $B_{0}=\left[\begin{array}{c} 0\\ I \end{array}\right]$, $C_{0}=\left[\begin{array}{cc} C_{h} & I \end{array}\right]$, $H=\left[\begin{array}{c} H_{1}\\ H_{2} \end{array}\right]$.

The error analysis, passivity, and stability analysis of the sliding mode observer can be seen in [35].

Therefore, it can be inferred that:

(23) $$∥\hat{\eta }-\eta ∥\leq B_{1},$$

(24) $$∥\hat{v}-v∥\leq B_{2}.$$

### 3.2. Design of Finite-Time Convergent Disturbance Observer

In this part, a finite-time convergent disturbance observer is designed to estimate unknown time-varying low-frequency disturbances.

Fundamentally, let instrumental Variable $p=M_{0}v$ and $\hat{p}$ be the estimated value of *p*. In addition, $\tilde{p}$ is defined as:

(25) $$\tilde{p}=p-\hat{p}.$$

The design of auxiliary variables is as follows:

(26) $$\dot{\tilde{p}}=-D_{1}\tilde{p}-D_{2}{\left|\tilde{p}\right|}^{\gamma }sign\left(\tilde{p}\right)-\bar{d}sign\left(\tilde{p}\right)-d.$$

According to (7), (25), (26), taking the derivative of $\hat{p}$

(27) $$\begin{array}{cc} \dot{\hat{p}} & =\dot{\tilde{p}}+\dot{p}\\ \; & =-D_{0}v+\tau +\Delta -D_{1}\tilde{p}-D_{2}{\left|\tilde{p}\right|}^{\gamma }sign\left(\tilde{p}\right)-\bar{d}sign\left(\tilde{p}\right), \end{array}$$

where $D_{1}=diag\left\{D_{11},D_{12},D_{13}\right\}$, $D_{2}=diag\left\{D_{21},D_{22},D_{23}\right\}$ represent finite-time convergent disturbance observer (FCDO) parameter matrices, $D_{1i}$ and $D_{2i}\left(i=1,2,3\right)$ are positive values. $\bar{d}$ is disturbance upper bound. 0<γ<1.

Assuming the estimation of *d* is $\hat{d}$ and the disturbance observer was previously involved in (27):

(28) $$\begin{array}{cc} \hat{d} & =\dot{\hat{p}}+D_{0}v-\tau -\Delta \\ \; & =-D_{1}\tilde{p}-D_{2}{\left|\tilde{p}\right|}^{\gamma }sign\left(\tilde{p}\right)-\bar{d}sign\left(\tilde{p}\right). \end{array}$$

**Theorem** **1.** *Based on the DP ship model Equation (7), FCDO can be derived from (25)–(28) with the estimated error* $\tilde{d}=d-\hat{d}$ *converging in finite time.*

**Proof of Theorem 1.**  Design the Lyapunov function as follows:

(29) $$\begin{array}{c} V_{do}=\frac{1}{2}{\tilde{p}}^{T}\tilde{p}. \end{array}$$

Using (26), the time derivative of $V_{do}$ is

(30) $$\begin{array}{cc} {\dot{V}}_{do} & ={\tilde{p}}^{T}\left(-D_{1}\tilde{p}-D_{2}{\left|\tilde{p}\right|}^{\gamma }sign\left(\tilde{p}\right)-\bar{d}sign\left(\tilde{p}\right)-d\right)\\ \; & \leq -2D_{1\min}V_{do}-2^{\gamma +1}D_{2\min}{V_{do}}^{\frac{\gamma +1}{2}}. \end{array}$$

where $D_{1\min}=\min\left\{D_{11},D_{12},D_{13}\right\}$ and $D_{2\min}=\min\left\{D_{21},D_{22},D_{23}\right\}$.

According to Lemma 1, the convergence of error $\tilde{p}$ to 0 in finite time is proved.

Synthesize Formulas (25)–(27):

(31) $$\begin{array}{cc} \tilde{d} & =d-\hat{d}\\ \; & =M_{0}\dot{v}+D_{0}v-\tau -\Delta +D_{1}\tilde{p}+D_{2}{\left|\tilde{p}\right|}^{\gamma }sign\left(\tilde{p}\right)+\bar{d}sign\left(\tilde{p}\right)\\ \; & =\dot{p}-\dot{\hat{p}}\\ \; & =-\dot{\tilde{p}}. \end{array}$$

Since the $\tilde{p}$ converges in finite time, the estimation error $\tilde{d}$ converges to 0, and the estimated value $\hat{d}$ is approximate to the uncertain disturbance *d* in finite time. 0<γ<1, $D_{2i}\left(i=1,2,3\right)$ are positive numbers, and $V_{do}$ is positive-definite, so,

(32) $$2^{\gamma +1}D_{2\min}{V_{do}}^{\frac{\gamma +1}{2}}>0.$$

According to (30) and (32):

(33) $$\begin{array}{cc} {\dot{V}}_{do} & \leq -2D_{1\min}V_{do}-2^{\gamma +1}D_{2\min}{V_{do}}^{\frac{\gamma +1}{2}}\\ & \leq -2D_{1\min}V_{do}. \end{array}$$

Therefore, Theorem 1 proposed below is available.

□

### 3.3. DP Adaptive Neural Output Feedback Control Design

The following error variables are defined:

(34) $$S_{1}=\eta -\eta_{d},$$

(35) $$S_{2}=v-\alpha_{1},$$

(36) $$S_{3}=\tau -\beta_{1},$$

where $\eta_{d}={\left[x_{d},y_{d},\psi_{d}\right]}^{T}$ is the expected ship position and heading. $\alpha_{1}\in R^{3}$ and $\beta_{1}\in R^{3}$ are intermediate control vectors that will be designed in the subsequent controller design process.

According to (7) and (34):

(37) $${\dot{S}}_{1}=R\left(\psi \right)v.$$

Define $\alpha_{1}\in R^{3\times 3}$

(38) $$\alpha_{1}=-R^{T}\left(\psi \right)k_{1}S_{1},$$

where $k_{1}={k_{1}}^{T}\in R^{3\times 3}$ is the positive definite parameter matrix to be designed.

According to (7), the derivation of (35):

(39) $$M_{0}{\dot{S}}_{2}=M_{0}\dot{v}-M_{0}{\dot{\alpha }}_{1}=-D_{0}\;v+\tau +d+\Delta -M_{0}{\dot{\alpha }}_{1}.$$

Define $\alpha_{2}\in R^{3\times 3}$:

(40) $$\alpha_{2}=-k_{2}S_{2}+D_{0}v+M_{0}{\dot{\alpha }}_{1}-R^{T}\left(\psi \right)S_{1}-\hat{d}-\Delta ,$$

where $k_{2}={k_{2}}^{T}\in R^{3\times 3}$ is the positive definite parameter matrix to be designed.

Define

(41) $$\begin{array}{cc} L(Z) & =\Delta +R^{T}\left(\psi \right)S_{1}-M_{0}{\dot{\alpha }}_{1}\\ \; & =-\Delta M\dot{v}-\Delta Dv+R^{T}\left(\psi \right)S_{1}-M_{0}{\dot{\alpha }}_{1}, \end{array}$$

where $Z={\left[{\begin{array}{ccc} {\dot{v}}^{T} & v^{T} & S_{1} \end{array}}^{T}\right]}^{T}$.

According to $∥R(\psi )∥=1$ and (41):

(42) $$\begin{array}{c} ∥L(Z)∥\leq ∥\Delta M∥∥\dot{v}∥+∥\Delta D∥∥v∥+∥S_{1}∥+∥M_{0}∥∥{\dot{\alpha }}_{1}∥\\ \;\leq \theta \overset{⌢}{\;\varphi }\left(Z\right), \end{array}$$

where

(43) $$\overset{⌢}{\;\varphi }(Z)=\dot{v}+∥v∥+∥S_{1}∥+1$$

(44) $$\theta =\max\left\{∥\Delta M∥,∥\Delta D∥,∥M_{0}∥,∥{\dot{\alpha }}_{1}∥,1\right\}.$$

Define

(45) $$\beta_{1}=-k_{2}S_{2}+D_{0}v-c\hat{\theta }{\overset{⌢}{\;\varphi }}^{2}(Z)S_{2}-\hat{d}$$

The adaptive law is

(46) $$\begin{array}{c} \dot{\hat{\theta }}=c{\overset{⌢}{\;\varphi }}^{2}(Z){∥S_{2}∥}^{2}-\sigma \hat{\theta },\hat{\theta }(0)\geq 0, \end{array}$$

where c>0 and σ>0 are design parameters and need to satisfy:

(47) $$c{\overset{⌢}{\;\varphi }}^{2}\left(Z\right)∥S_{2}∥\geq 1$$

Using (40), (42), and (45), it can be obtained:

(48) $$\left\{\begin{array}{c} ∥L(Z)∥\leq \theta \overset{⌢}{\;\varphi }(Z)\leq c{\overset{⌢}{\;\varphi }}^{2}(Z)∥S_{2}∥\\ {S_{2}}^{T}L(Z)\leq ∥S_{2}∥∥L(Z)∥\leq c\theta {\overset{⌢}{\;\varphi }}^{2}(Z)∥S_{2}∥\leq {S_{2}}^{T}c\theta {\overset{⌢}{\;\varphi }}^{2}(Z)S_{2}\\ {S_{2}}^{T}\beta_{1}\leq {S_{2}}^{T}\alpha_{1} \end{array}\right..$$

Deriving (36) from (7) and using $\Delta \tau =\tau_{p}-\tau_{c}$:

(49) $$\begin{array}{cc} {A_{\mathrm{tr}}}^{-1}{\dot{S}}_{3} & ={A_{tr}}^{-1}\dot{\tau }-{A_{tr}}^{-1}{\dot{\beta }}_{1}\\ \; & =-\tau +\tau_{c}+\Delta \tau -{A_{\mathrm{tr}}}^{-1}{\dot{\beta }}_{1}. \end{array}$$

An auxiliary dynamic system is introduced.

(50) $$\begin{array}{c} {\dot{\xi }}_{1}=\left\{\begin{array}{c} -k_{\xi }\xi_{1}-\frac{\sum_{i=1}^{3}\left|s_{3,i}\Delta \tau_{i}\right|+0.5\Delta \tau^{T}\Delta \tau }{{∥\xi_{1}∥}^{2}}\xi_{1}+\Delta \tau ,\;∥\xi_{1}∥\geq \xi_{0}\\ 0_{3\times 1},\;\;\;\;\;\;∥\xi_{1}∥<\xi_{0} \end{array}\right., \end{array}$$

where $\xi_{1}={\left[\xi_{1},\xi_{2},\xi_{3}\right]}^{T}$ is the state variable of the auxiliary dynamic system. $k_{\xi }={k_{\xi }}^{T}\in R^{3\times 3}$ is positive definite design matrix, and $\xi_{0}>0$. When $∥\xi_{1}∥<\xi_{0}$ taking $\dot{\xi_{1}}=0_{3\times 1}$ can avoid singular problems. The adaptive neural state feedback law for DP is given below:

(51) $$\begin{array}{c} \tau_{c0}=-k_{3}S_{3}+\tau +{A_{tr}}^{-1}\beta_{1}+k_{\xi }\xi_{1}-S_{2}, \end{array}$$

where $k_{3}={k_{3}}^{T}\in R^{3}$ and $k_{\xi }={k_{\xi }}^{T}\in R^{3}$ are positive definite design matrices.

$\hat{\eta }$ and $\hat{v}$ obtained by the sliding mode observer are used in the next controller design to replace η and *v*. Define the new error variable as

(52) $${\hat{S}}_{1}=\hat{\eta }-\eta_{d},$$

(53) $${\hat{S}}_{2}=\hat{v}-{\hat{\alpha }}_{1},$$

(54) $${\hat{S}}_{3}=\tau -{\hat{\beta }}_{1},$$

where

(55) $${\hat{\alpha }}_{1}=-R^{T}\left(\psi \right)k_{1}{\hat{S}}_{1},$$

(56) $${\hat{\beta }}_{1}=-k_{2}{\hat{S}}_{2}+D_{0}\hat{v}-c\hat{\theta }{\overset{⌢}{\;\varphi }}^{2}(Z){\hat{S}}_{2}-\hat{d}$$

The adaptive law is

(57) $$\dot{\hat{\theta }}=c{\overset{⌢}{\;\varphi }}^{2}(\hat{Z}){∥S_{2}∥}^{2}-\sigma \hat{\theta },\hat{\theta }(0)\geq 0.$$

At this time, the input saturation auxiliary dynamic system is changed to (58).

(58) $${\dot{\xi }}_{1}=\left\{\begin{array}{c} -k_{\xi }\xi_{1}-\frac{\sum_{i=1}^{3}\left|{\hat{s}}_{3,i}\Delta \tau_{i}\right|+0.5\Delta \tau^{T}\Delta \tau }{{∥\xi_{1}∥}^{2}}\xi_{1}+\Delta \tau ,\;∥\xi_{1}∥\geq \xi_{0}\\ 0_{3\times 1},\;\;\;\;\;\;∥\xi_{1}∥<\xi_{0} \end{array}\right..$$

The new error variable is

(59) $${\tilde{S}}_{1}={\hat{S}}_{1}-S_{1},$$

(60) $${\tilde{S}}_{2}={\hat{S}}_{2}-S_{2},$$

(61) $${\tilde{S}}_{3}={\hat{S}}_{3}-S_{3}.$$

According to (34) and (52):

(62) $$\begin{array}{cc} {\tilde{S}}_{1}^{T}{\tilde{S}}_{1} & ={∥{\hat{S}}_{1}-S_{1}∥}^{2}\\ \; & =∥\hat{\eta }-\eta ∥\\ \; & \leq {B_{1}}^{2}. \end{array}$$

According to (38), (55), (62), and R(ψ)=1:

(63) $$\begin{array}{cc} ∥{\hat{\alpha }}_{1}-\alpha_{1}∥ & =∥-R^{T}\left(\psi \right)k_{1}{\hat{S}}_{1}+R^{T}\left(\psi \right)k_{1}S_{1}∥\\ \; & =∥k_{1}∥∥\hat{\eta }-\eta ∥\\ \; & \leq {B_{1}}^{2}. \end{array}$$

According to (34), (35), (38), (52), (53), (55), (59), and $∥R(\psi )∥=1$:

(64) $$\begin{array}{cc} {\tilde{S}}_{2}^{T}{\tilde{S}}_{2}\; & ={∥\hat{v}-v-{\hat{\alpha }}_{1}+\alpha_{1}∥}^{2}\\ \; & \leq {\left(B_{2}+∥k_{1}∥B_{1}\right)}^{2}. \end{array}$$

According to (62):

(65) $$\begin{array}{c} \overset{⌢}{\;\varphi } \end{array}\left(\hat{Z}\right)=∥\dot{\hat{v}}∥+∥\hat{v}∥+∥{\hat{S}}_{1}∥+1.$$

According to (62) and (65):

(66) $$\begin{array}{c} \overset{⌢}{\;\varphi } \end{array}\left(\hat{Z}\right)-\overset{⌢}{\;\varphi }(Z)\leq ∥\dot{\hat{v}}-\dot{v}∥+∥\hat{v}-v∥+∥{\hat{S}}_{1}-S_{1}∥$$

$\hat{v}$ and *v* are continuously derivable and satisfy the Lipschitz continuity condition. Therefore, $\left(\hat{v}-v\right)$ is continuously derivable and satisfies the Lipschitz continuity condition, i.e., $\left(\dot{\hat{v}}-\dot{v}\right)$ is bounded, then

(67) $$∥\dot{\hat{v}}-\dot{v}∥\leq B_{3},$$

where $B_{3}$ is the normal number.

According to (34), (52), (66), and (67):

(68) $$\begin{array}{c} \overset{⌢}{\;\varphi } \end{array}\left(\hat{Z}\right)-\overset{⌢}{\;\varphi }\left(Z\right)\leq B_{3}+B_{2}+B_{1}$$

(69) $$\begin{array}{cc} ∥{\hat{S}}_{3}-S_{3}∥ & \leq B_{4}, \end{array}$$

(70) $$\begin{array}{cc} ∥{\dot{\hat{\beta }}}_{1}-{\hat{\beta }}_{1}∥ & \leq B_{5}, \end{array}$$

where $B_{4}$ and $B_{5}$ are normal numbers.

According to (61) and (69):

(71) $$\begin{array}{c} {\tilde{S}}_{3}^{T}{\tilde{S}}_{3}={∥{\hat{S}}_{3}-S_{3}∥}^{2}\leq {B_{4}}^{2} \end{array}.$$

Therefore, the DP adaptive neural output feedback control law based on sliding mode observer design is

(72) $$\tau_{c}=-k_{3}{\hat{S}}_{3}+\tau +{A_{tr}}^{-1}{\hat{\beta }}_{1}+k_{\xi }\xi_{1}-{\hat{S}}_{2}.$$

## 4. System Stability Analysis

The design Lyapunov function *V*

(73) $$\begin{array}{cc} V= & \frac{1}{2}{S_{1}}^{T}S_{1}+\frac{1}{2}{S_{2}}^{T}M_{0}S_{2}+\frac{1}{2}{S_{3}}^{T}{A_{tr}}^{-1}S_{3}+\frac{1}{2g}{\left(\theta -g\hat{\theta }\right)}^{2}+\frac{1}{2}{\xi_{1}}^{T}\xi_{1}+\frac{1}{2}\tilde{p^{T}}\tilde{p}. \end{array}$$

Derivation of (73)

(74) $$\begin{array}{cc} \dot{V}= & {S_{1}}^{T}{\dot{S}}_{1}+{S_{2}}^{T}M_{0}{\dot{S}}_{2}+{S_{3}}^{T}{A_{tr}}^{-1}{\dot{S}}_{3}+\left(\theta -g\hat{\theta }\right)\dot{\hat{\theta }}+{\xi_{1}}^{T}\xi_{1}+{\tilde{p}}^{T}\dot{\tilde{p}}. \end{array}$$

According to (35)–(38), $∥R(\psi )∥=1$, and Young’s inequality:

(75) $$\begin{array}{cc} {S_{1}}^{T}{\dot{S}}_{1} & ={S_{1}}^{T}R\left(\psi \right)\left(S_{2}+\alpha_{1}\right)\\ \; & =-{S_{1}}^{T}k_{1}S_{1}+{S_{1}}^{T}R\left(\psi \right)S_{2}. \end{array}$$

According to (36), (39), (40), (48), $∥R(\psi )∥=1$, and Young’s inequality:

(76) $$\begin{array}{cc} {S_{2}}^{T}M_{0}{\dot{S}}_{2}\; & ={S_{2}}^{T}\left(-k_{2}S_{2}-R^{T}\left(\psi \right)S_{1}-\hat{d}-\alpha_{2}+\tau +d\right)\\ \; & \leq -{S_{2}}^{T}k_{2}S_{2}-\frac{1}{2}{S_{1}}^{T}S_{1}+\frac{1}{2}{S_{2}}^{T}S_{2}+\frac{1}{2}{S_{3}}^{T}S_{3}+\frac{1}{2}{\tilde{d}}^{T}\tilde{d} \end{array}$$

According to (49), (60), (61), (64), (70)–(72), and Young’s inequality:

(77) $$\begin{array}{cc} {S_{3}}^{T}{A_{tr}}^{-1}{\dot{S}}_{3}=\; & {S_{3}}^{T}\left(-\tau +\tau_{c}+\Delta \tau -{A_{tr}}^{-1}{\dot{\beta }}_{1}\right)\\ \; & -{S_{3}}^{T}S_{2}+{S_{3}}^{T}\Delta \tau \\ \leq & -{S_{3}}^{T}k_{3}S_{3}+{S_{3}}^{T}\Delta \tau +\frac{5}{2}{S_{3}}^{T}S_{3}+\frac{1}{2}{S_{2}}^{T}S_{2}+\frac{1}{4}{∥k_{3}∥}^{2}+\frac{1}{4}{B_{4}}^{2}\\ \; & +\frac{1}{4}{∥{A_{tr}}^{-1}∥}^{2}+\frac{1}{4}{B_{5}}^{2}+\frac{1}{2}{\xi_{1}}^{T}{k_{\xi }}^{T}k_{\xi }\xi_{1}+\frac{1}{2}{\left(B_{2}+∥k_{1}∥B_{1}\right)}^{2}. \end{array}$$

Let $\tilde{\theta }=\theta -g\hat{\theta }$, according to (57):

(78) $$\begin{array}{c} -\left(\theta -g\hat{\theta }\right)\dot{\hat{\theta }}=-\tilde{\theta }(c{\overset{⌢}{\;\varphi }}^{2}\left(\hat{Z}\right)∥{\hat{S}}_{2}∥-\sigma \hat{\theta })\\ \leq \sigma \tilde{\theta }\hat{\theta } \end{array}.$$

According to $\tilde{\theta }=\theta -g\hat{\theta }$ and Young’s inequality:

(79) $$\begin{array}{cc} \tilde{\theta }\hat{\theta } & =\frac{1}{g}\tilde{\theta }\left(\theta -\tilde{\theta }\right)\\ \; & \leq -\frac{1}{2g}{\tilde{\theta }}^{2}+\frac{1}{2g}\theta^{2}, \end{array}$$

(80) $$\begin{array}{cc} -\left(\theta -g\hat{\theta }\right)\dot{\hat{\theta }} & \leq -\frac{1}{2g}\sigma {\tilde{\theta }}^{2}+\frac{1}{2g}\sigma \theta^{2}\\ \; & \leq -\frac{1}{8g}\sigma {\left|\tilde{\theta }\right|}^{2}+\frac{1}{8g}\sigma +\frac{1}{2g}\sigma \theta^{2}. \end{array}$$

When $∥\xi_{1}∥\geq \xi_{0}$, according to (58) and Young’s inequality:

(81) $$\begin{array}{cc} {\xi_{1}}^{T}\dot{\xi_{1}} & =-{\xi_{1}}^{T}k_{\xi }\xi_{1}-\sum_{i=1}^{3}\left|{\hat{S}}_{3,i}\Delta \tau_{i}\right|-\frac{1}{2}\Delta \tau^{T}\Delta \tau +{\xi_{1}}^{T}\Delta \tau \\ \; & \leq -{\xi_{1}}^{T}k_{\xi }\xi_{1}-\sum_{i=1}^{3}\left|{\hat{S}}_{3,i}\Delta \tau_{i}\right|+\frac{1}{2}{\xi_{1}}^{T}\xi_{1}. \end{array}$$

When $∥\xi_{1}∥<\xi_{0}$, according to (58) and Young’s inequality:

(82) $$\begin{array}{cc} {\xi_{1}}^{T}\dot{\xi_{1}} & =0, \end{array}$$

(83) $$\begin{array}{cc} \frac{1}{2}{\xi_{1}}^{T}{k_{\xi }}^{T}k_{\xi }\xi_{1} & ={\xi_{1}}^{T}{k_{\xi }}^{T}k_{\xi }\xi_{1}-\frac{1}{2}{\xi_{1}}^{T}{k_{\xi }}^{T}k_{\xi }\xi_{1}\\ \; & <-\frac{1}{2}{\xi_{1}}^{T}{k_{\xi }}^{T}k_{\xi }\xi_{1}+{\xi_{1}}^{2}∥{k_{\xi }}^{T}k_{\xi }∥, \end{array}$$

(84) $$\begin{array}{cc} {S_{3}}^{T}\Delta \tau & \leq \frac{1}{2}{S_{3}}^{T}S_{3}+\frac{1}{2}{∥\Delta \tau ∥}^{2} \end{array}.$$

In the case of $∥\xi_{1}∥\geq \xi_{0}$, substituting (75), (76), (77), (80), and (81) into (74):

(85) $$\begin{array}{cc} \dot{V}\leq & -{S_{1}}^{T}k_{1}S_{1}-{S_{2}}^{T}k_{2}S_{2}-{S_{3}}^{T}k_{3}S_{3}+{S_{3}}^{T}\Delta \tau \\ \; & +\sum_{i=1}^{3}\left|{\hat{S}}_{3,i}\Delta \tau_{i}\right|+\frac{3}{2}{S_{2}}^{T}S_{2}+3{S_{3}}^{T}S_{3}+\frac{1}{2}{\tilde{d}}^{T}\tilde{d}-{\tilde{p}}^{T}D_{1}\tilde{p}\\ \; & -{\xi_{1}}^{T}\left(k_{\xi }-\frac{1}{2}{k_{\xi }}^{T}k_{\xi }-\frac{1}{2}I_{3\times 3}\right)\xi_{1}+\frac{1}{2}{\left(B_{2}+∥k_{1}∥B_{1}\right)}^{2}+\frac{1}{4}B_{4}\\ \; & +\frac{1}{4}{∥k_{3}∥}^{2}+\frac{1}{4}∥{A_{tr}}^{-1}∥+\frac{1}{4}B_{5}-\frac{1}{8g}\sigma {\left|\tilde{\theta }\right|}^{2}+\frac{1}{8g}\sigma +\frac{1}{2g}\sigma \theta^{2}. \end{array}$$

In (85), according to (61), (71), and Young’s inequality:

(86) $$\begin{array}{cc} {S_{3}}^{T}\Delta \tau -\sum_{i=3}^{3}\left|{\hat{S}}_{3,i}\Delta \tau_{i}\right| & ={S_{3}}^{T}\Delta \tau -{{\hat{S}}_{3}}^{T}\Delta \tau \\ \; & \leq \frac{1}{2}{B_{4}}^{2}+\frac{1}{2}{\gamma_{1}}^{2}, \end{array}$$

where $\gamma_{1}$ denotes the upper bound of $∥\Delta \tau ∥$.

Substituting (86) into (85):

(87) $$\begin{array}{cc} \dot{V}\leq & -{S_{1}}^{T}k_{1}S_{1}-{S_{2}}^{T}\left(k_{2}-\frac{3}{2}I_{3\times 3}\right)S_{2}-{S_{3}}^{T}\left(k_{3}-\frac{3}{2}I_{3\times 3}\right)S_{3}-\frac{1}{8g}\sigma {\left|\tilde{\theta }\right|}^{2}\\ \; & -{\xi_{1}}^{T}\left(k_{\xi }-\frac{1}{2}{k_{\xi }}^{T}k_{\xi }-\frac{1}{2}I_{3\times 3}\right)\xi_{1}-{\tilde{p}}^{T}D_{1}\tilde{p}+\frac{1}{2}{∥\tilde{d}∥}^{2}+\frac{1}{4}{∥{A_{tr}}^{-1}∥}^{2}+\frac{1}{4}B_{5}\\ \; & +\frac{1}{8g}\sigma +\frac{1}{2g}\sigma \theta^{2}+\frac{3}{4}{B_{4}}^{2}+\frac{1}{2}{\gamma_{1}}^{2}+\frac{1}{2}{\left(B_{2}+∥k_{1}∥B_{1}\right)}^{2}+\frac{1}{4}{∥k_{3}∥}^{2}\\ \; & \leq -2\mu_{1}V+C_{1}, \end{array}$$

where

$$\begin{array}{cc} \mu_{1}=\; & \min\{\lambda_{\min}\left(k_{1}\right),\lambda_{\min}\left[\left(k_{2}-\frac{3}{2}I_{3\times 3}\right){M_{0}}^{-1}\right],\lambda_{\min}\left[\left(k_{3}-3I_{3\times 3}\right)A_{tr}\right],\frac{\sigma }{8},\\ \; & \lambda_{\min}\left(k_{\xi }-\frac{1}{2}{k_{\xi }}^{T}k_{\xi }-\frac{1}{2}I_{3\times 3}\right),\lambda_{\min}\left(D_{1}\right)\}, \end{array}$$

and

$$\begin{array}{cc} C_{1}= & \;\frac{1}{2}{∥\tilde{d}∥}^{2}+\frac{1}{4}{∥{A_{tr}}^{-1}∥}^{2}+\frac{1}{4}{B_{5}}^{2}+\frac{1}{8g}\sigma +\frac{1}{2g}\sigma \theta^{2}+\frac{3}{4}{B_{4}}^{2}+\frac{1}{2}{\gamma_{1}}^{2}\\ \; & +\frac{1}{2}{\left(B_{2}+∥k_{1}∥B_{1}\right)}^{2}+\frac{1}{4}{∥k_{3}∥}^{2}. \end{array}$$

The design matrices need to meet:

(88) $$\lambda_{\min}\left(k_{1}\right)>0,$$

(89) $$\lambda_{\min}\left(k_{2}\right)>\frac{3}{2},$$

(90) $$\lambda_{\min}\left(k_{3}\right)>3,$$

(91) $$\lambda_{\min}\left(k_{\xi }-\frac{1}{2}{k_{\xi }}^{T}k_{\xi }-\frac{1}{2}I_{3\times 3}\right)>0.$$

In the case of $∥\xi_{1}∥<\xi_{0}$, substituting (75)–(77), (80), and (82)–(84) into (74), we organize and get

(92) $$\begin{array}{cc} \dot{V}\leq & -{S_{1}}^{T}k_{1}S_{1}-{S_{2}}^{T}\left(k_{2}-\frac{3}{2}I_{3\times 3}\right)S_{2}-{S_{3}}^{T}\left(k_{3}-\frac{7}{2}I_{3\times 3}\right)S_{3}-\frac{1}{8g}\sigma {\left|\tilde{\theta }\right|}^{2}\\ \; & \;\;\;\;\;-\frac{1}{2}{\xi_{1}}^{T}{k_{\xi }}^{T}k_{\xi }\xi_{1}-{\tilde{p}}^{T}D_{1}\tilde{p}+\frac{1}{2}{∥\tilde{d}∥}^{2}+\frac{1}{2}{\left(B_{2}+∥k_{1}∥B_{1}\right)}^{2}+\frac{1}{4}{B_{4}}^{2}\\ \; & \;\;\;\;\;+\frac{1}{4}{∥k_{3}∥}^{2}+\frac{1}{4}{∥{A_{tr}}^{-1}∥}^{2}+\frac{1}{4}{B_{5}}^{2}+\frac{1}{8g}\sigma +\frac{1}{2g}\sigma \theta^{2}+{\xi_{0}}^{2}∥{k_{\xi }}^{T}k_{\xi }∥+\frac{1}{2}{\gamma_{1}}^{2}\\ \; & \leq -2\mu_{2}V+C_{2}, \end{array}$$

where

$$\begin{array}{cc} \mu_{2}=\; & \min\{\lambda_{\min}\left(k_{1}\right),\lambda_{\min}\left[\left(k_{2}-\frac{3}{2}I_{3\times 3}\right){M_{0}}^{-1}\right],\lambda_{\min}\left[\left(k_{3}-\frac{7}{2}I_{3\times 3}\right)A_{tr}\right],\frac{\sigma }{8},\\ \; & \lambda_{\min}\left({k_{\xi }}^{T}k_{\xi }\right),\lambda_{\min}\left(D_{1}\right)\}, \end{array}$$

and

$$\begin{array}{cc} C_{2}= & \;\frac{1}{2}{∥\tilde{d}∥}^{2}+\frac{1}{2}{\left(B_{2}+∥k_{1}∥B_{1}\right)}^{2}+\frac{1}{4}{B_{4}}^{2}+\frac{1}{4}{B_{5}}^{2}+\frac{1}{2}{\gamma_{1}}^{2}+\frac{1}{4}{∥{A_{tr}}^{-1}∥}^{2}+\frac{1}{4}{∥k_{3}∥}^{2}\\ \; & +\frac{1}{8g}\sigma +\frac{1}{2g}\sigma \theta^{2}+{\xi_{0}}^{2}∥{k_{\xi }}^{T}k_{\xi }∥. \end{array}$$

The design matrices need to meet:

(93) $$\lambda_{\min}\left(k_{1}\right)>0,$$

(94) $$\lambda_{\min}\left(k_{2}\right)>\frac{3}{2},$$

(95) $$\lambda_{\min}\left(k_{3}\right)>\frac{7}{2},$$

(96) $$\lambda_{\min}\left({k_{\xi }}^{T}k_{\xi }\right)>0.$$

Combining (87) with (92), this yields:

(97) $$\begin{array}{c} \dot{V}\leq -2\mu V+C, \end{array}$$

where $\mu =\min\left\{\mu_{1},\mu_{2}\right\}$, $C=\max\left\{C_{1},C_{2}\right\}$.

For DP ships (7) considering high-frequency disturbances, input saturation, model uncertainty, and thruster system dynamics, the adaptive output feedback control law (74) based on sliding mode observers (14)–(18) designed in this chapter can dynamically locate control targets. Especially, by designing appropriate parameter matrices, all control signals in the closed-loop control system designed in this paper can be bounded.

## 5. Simulation and Comparison Studies

In this section, the proposed minimal-sensor control framework based on multi-observer fusion is analyzed and validated through numerical simulations implemented in the SimuNPS platform. The proposed approach is evaluated in terms of its state estimation accuracy with reduced sensor dependency and the performance of a multi-observer fusion strategy. The simulation studies are conducted on the supply ship Northern Clipper, which has a length of 76.2m and a mass of $4.591\times 10^{6}\;\mathrm{kg}$. The vessel parameters are adopted from [5].

$$\begin{array}{cccc} M_{0} & =\left[\begin{array}{ccc} 5.3122\times 10^{6} & 0 & 0\\ 0 & 8.2831\times 10^{6} & 0\\ 0 & 0 & 3.7454\times 10^{9} \end{array}\right],A_{tr} & =\left[\begin{array}{ccc} 0.2 & 0 & 0\\ 0 & 0.2 & 0\\ 0 & 0 & 0.2 \end{array}\right] & \\ D_{0} & =\left[\begin{array}{ccc} 5.0242\times 10^{4} & 0 & 0\\ 0 & 2.7229\times 10^{5} & -4.3933\times 10^{6}\\ 0 & -4.3933\times 10^{6} & 4.1894\times 10^{8} \end{array}\right] & \end{array}$$

The maximum amplitude of the thruster of the supply ship is shown in Table 1.

The scene parameters are set as: $b\left(0\right)={\left[20\;\mathrm{kN},20\;\mathrm{kN},20\;\mathrm{kN}\right]}^{T}$, $T=diag(10^{3},10^{3},10^{3})$, $\Psi =diag(3\times 10^{4},3\times 10^{3},3\times 10^{5})$, $\omega_{pi}=0.97\;\mathrm{rad}/s$, $\zeta_{i}=0.6083$. Its uncertainty matrix is $\Delta M=33\%M_{0}$, $\Delta D=11\%D_{0}$.

The desired ship position and heading are $\eta_{d}={\left[0\;m,0\;m,0^{\circ }\right]}^{T}$, and the initial states are $\eta \left(0\right)={\left[50\;m,50\;m,10^{\circ }\right]}^{T}$, $v\left(0\right)={\left[0\;m/s,0\;m/s,0^{\circ }/s\right]}^{T}$, $\tau \left(0\right)={\left[0,0,0\right]}^{T}$, $\xi \left(0\right)=[5\times 10^{4},5\times 10^{4},5\times 10^{4}{]}^{T}$, $\hat{\eta }\left(0\right)={\left[50\;m,50\;m,10^{\circ }\right]}^{T}$, $\hat{v}\left(0\right)={\left[0\;m/s,0\;m/s,0^{\circ }/s\right]}^{T}$, $\hat{p}\left(0\right)={\left[0,0,0\right]}^{T}$. Select the parameter in the simulation experiment of the sliding mode observer: $H_{1}={\left[diag\left(-2.0412,-2.0412,-2.0412\right),diag\left(1.764,1.764,1.764\right)\right]}^{T}$, $H_{2}=diag\left(1.1,1.1,1.1\right)$, $H_{3}=diag\left(0.01,0.01,0.01\right)$, $H_{4}=diag\left(0.1,0.1,0.1\right)$.

The design parameters are $D_{1}=diag\left(200,200,200\right)$, $D_{2}=diag\left(0.2,0.2,0.2\right)$, γ=0.6, $k_{1}=diag\left(0.1,0.1,0.1\right)$, $k_{2}=diag\left(5\times 10^{3},5\times 10^{3},5\times 10^{3}\right)$, $k_{3}=diag\left(5,5,5\right)$, $c=5\times 10^{5}$, $\sigma =1\times 10^{-6}$, $\xi_{0}=20$, and $K_{\xi }=diag\left(5,5,5\right)$.

### 5.1. Performance of the Sliding Mode Observer

The results show that the sliding mode observer can filter out the high-frequency motion of the ship and reconstruct the low-frequency motion.

**Figure 2:**
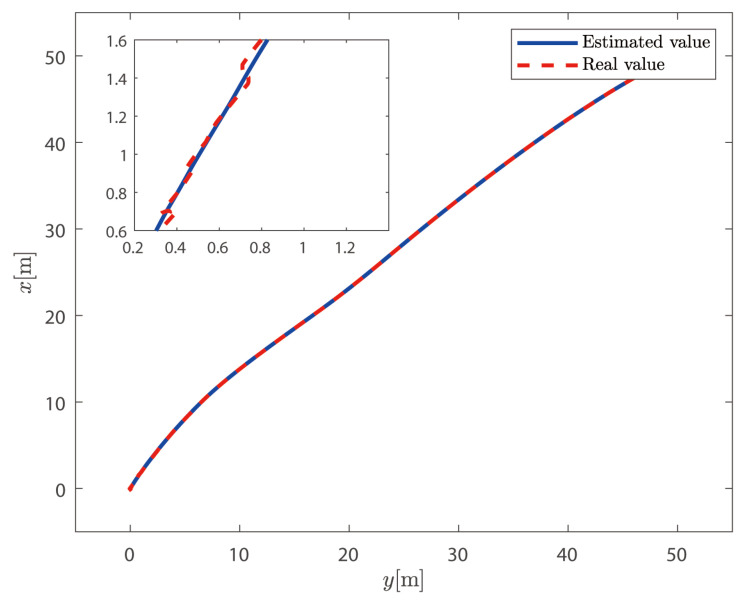
Horizontal motion trajectory of the ship.

**Figure 3:**
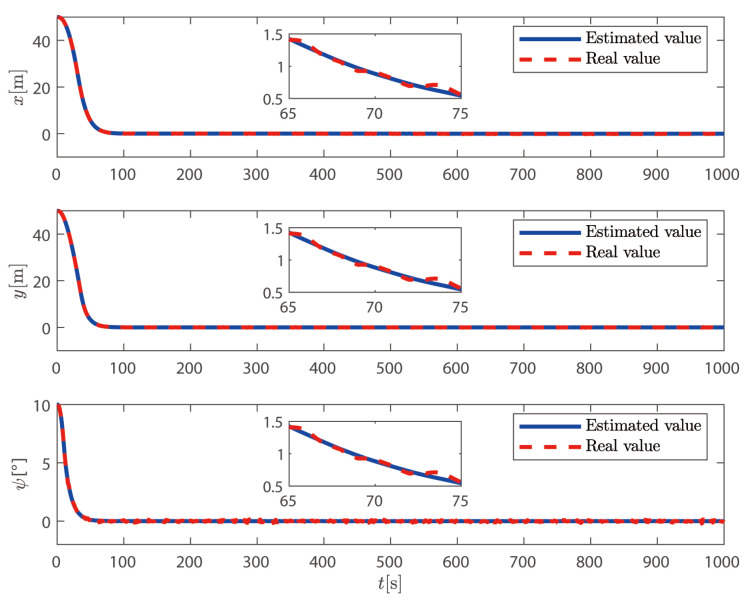
Ship position and heading angle.

**Figure 4:**
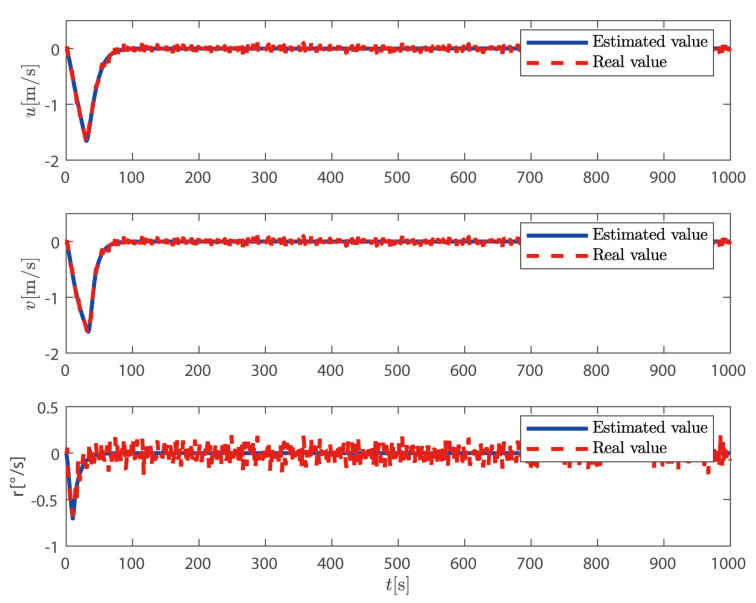
Ship velocity components.

It can be seen from Figure 2 and Figure 3 that, based on the high-low frequency linear superposition model of the ship, the sliding mode observer proposed in this article can filter out the high-frequency position and heading angle components of the ship and can reconstruct the low-frequency position and heading angle information. The ship can complete the DP operation well under the action of the controller proposed in this paper. It can be seen from Figure 4 that the sliding mode observer can effectively estimate the speed of the ship, and the speed of the ship changes uniformly within a certain range according to the trajectory of the ship. In summary, the sliding mode observer can effectively filter out the high-frequency information feedback and estimate the low-frequency position and speed information of the ship.

### 5.2. Performance of Output Feedback Controller Based on the Sliding Mode Observer

**Figure 5:**
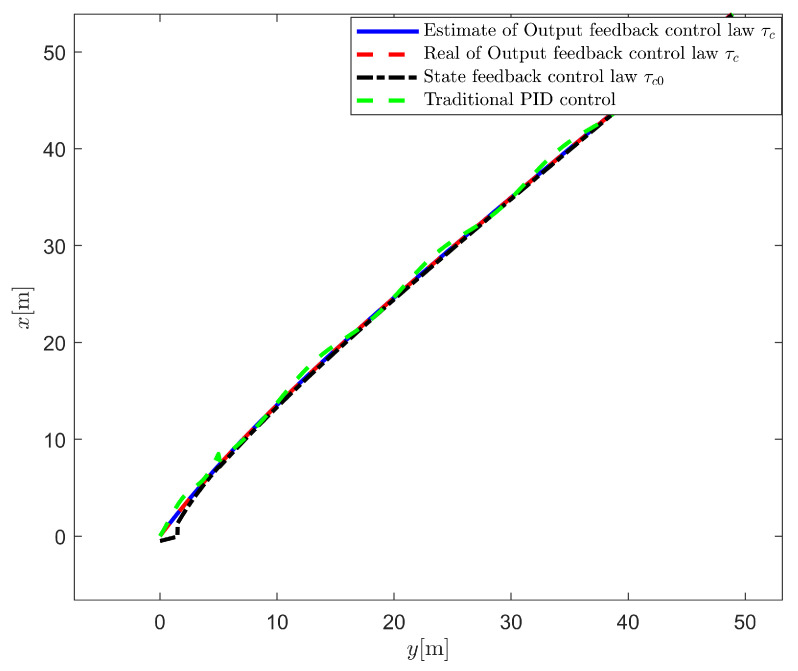
Comparative study of motion trajectories.

**Figure 6:**
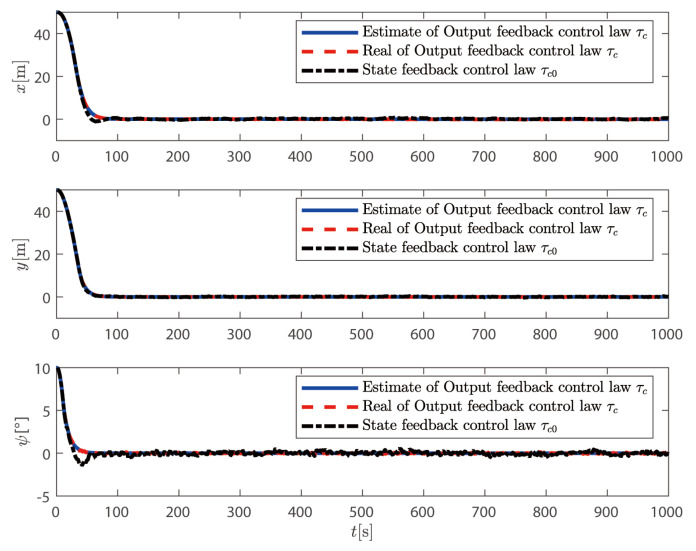
Output feedback vs state feedback: position and heading analysis.

**Figure 7:**
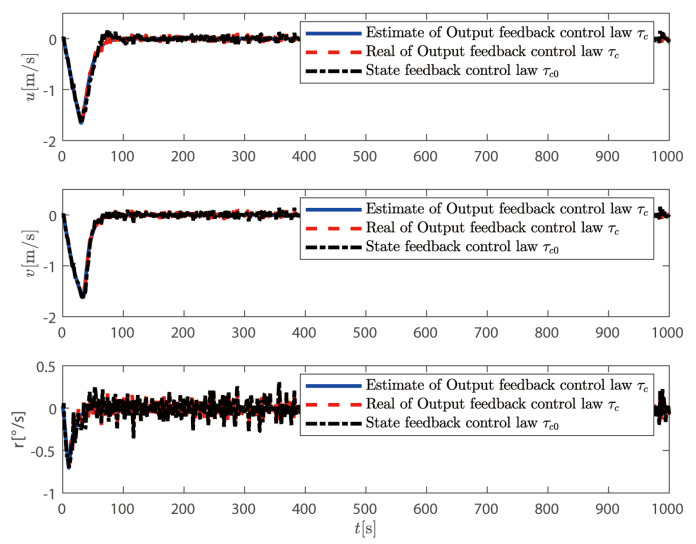
Velocity response comparison between control methods.

**Figure 8:**
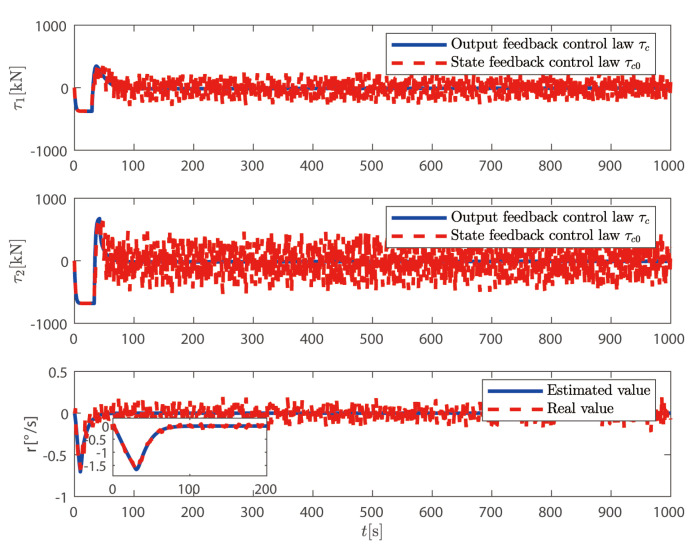
Control input comparison.

In this section, the output feedback control law $\tau_{c}$ proposed in (72) is compared with the state feedback control law $\tau_{0}$ proposed in (42). The simulation results are shown in Figure 5, Figure 6, Figure 7 and Figure 8.

As shown in Figure 5, we have implemented comparative simulations between our proposed control method, state feedback control, and traditional PID control. The comparative trajectories demonstrate that our proposed method exhibits better tracking performance and smaller steady-state errors compared to both state feedback and PID control, validating the effectiveness of our approach in reducing system conservatism.

From Figure 5 and Figure 6, it can be seen that although both control methods can achieve the desired position and heading angle, the ship under state feedback control exhibits larger high-frequency oscillations compared to the output feedback control, both in quantity and amplitude. Figure 6 shows that while the output feedback control produces some position overshoot, it demonstrates better overall performance than the state feedback control. As illustrated in Figure 7, the state feedback control generates more high-frequency components in ship speed compared to the output feedback approach. Figure 8 reveals that without filtering high-frequency motion states, the state feedback control allows these components to feed back into the controller, leading to unnecessary control actions against these high-frequency states and increased actuator wear. In contrast, our output feedback control effectively filters high-frequency motions, ensuring only low-frequency components are fed back to the controller, thus reducing actuator wear and extending system longevity.

### 5.3. Performance of Thruster System Dynamics

$\tau_{c}$ proposed above is compared with the control law without considering the dynamics of the thruster system proposed in Reference [36].

**Figure 9:**
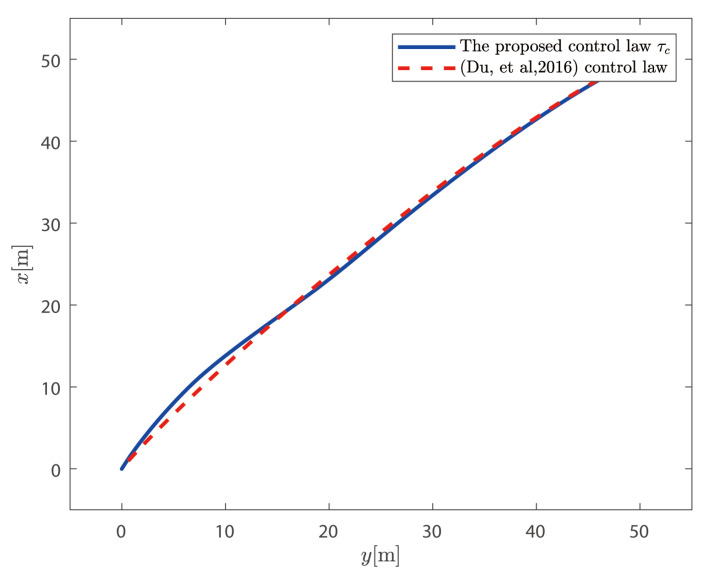
Horizontal motion trajectory comparison with control law from [36].

**Figure 10:**
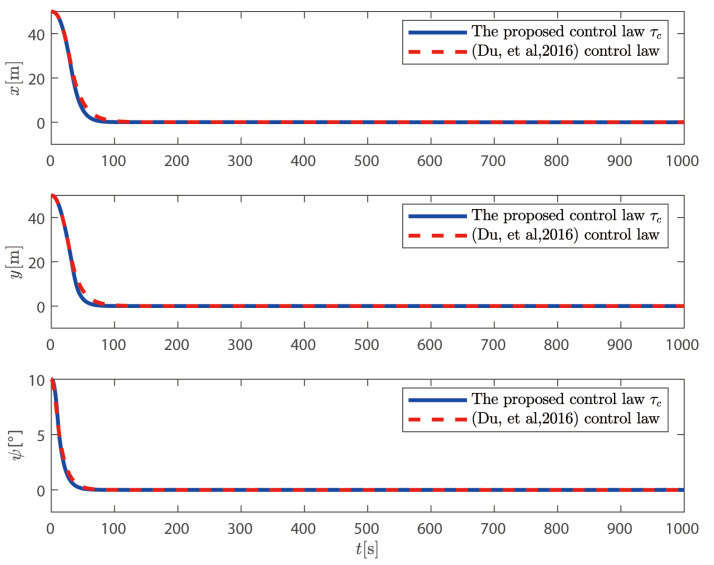
Position and heading response comparison with method in [36].

**Figure 11:**
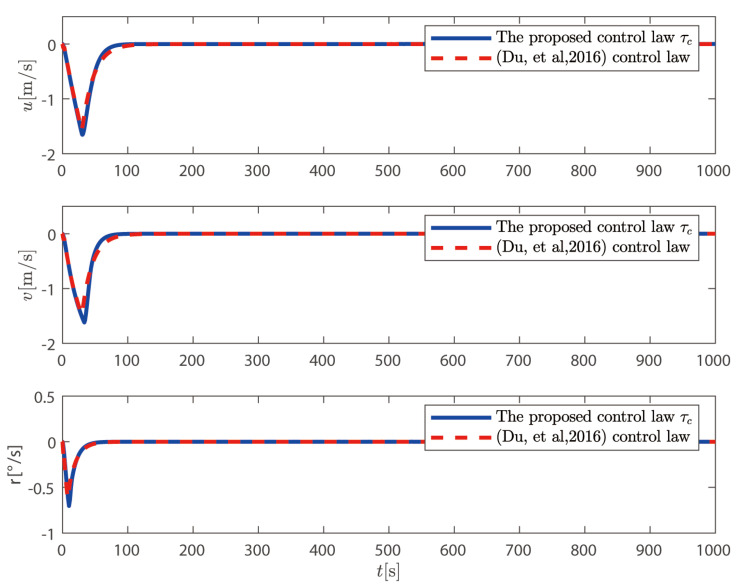
Velocity comparison with approach presented in [36].

It can be seen from Figure 9 and Figure 10 that the control law proposed in this paper can position the ship at the desired position and heading. It can also be seen from Figure 11 that the speed of the ship is bounded.

**Figure 12:**
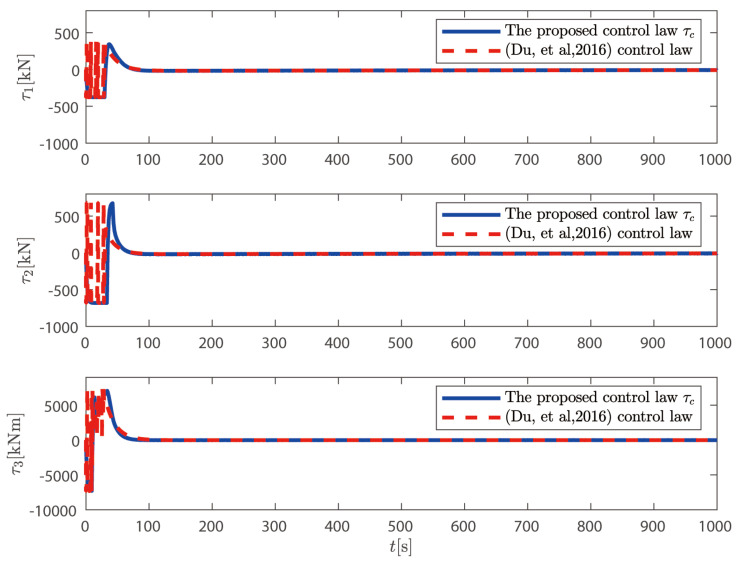
Thrust comparison with control method from [36].

**Figure 13:**
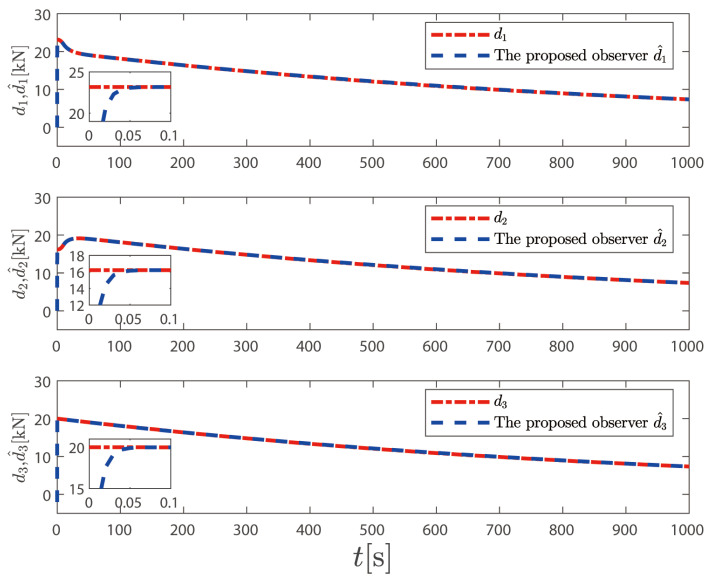
Estimated and true values of disturbance.

In Figure 12, the actual control variables generated by the control law proposed in this paper change smoothly and without mutation; the actual control signal designed in Reference [37] fluctuates greatly near the initial time, and there is a significant mutation. This shows that the control law proposed above is more in line with engineering practice than the control law in [37]. In Figure 13, we can observe that the FCDO can effectively track and estimate the unknown time-varying disturbances. The estimation errors converge to a small neighborhood of zero within a finite time, validating the theoretical analysis. The robust tracking performance is maintained throughout the simulation period despite the time-varying nature of the disturbances. This demonstrates the effectiveness of the proposed observer in handling unknown time-varying low-frequency disturbances in practical marine environments.

### 5.4. Advantages of Single Parameter Learning Neural Network

**Figure 14:**
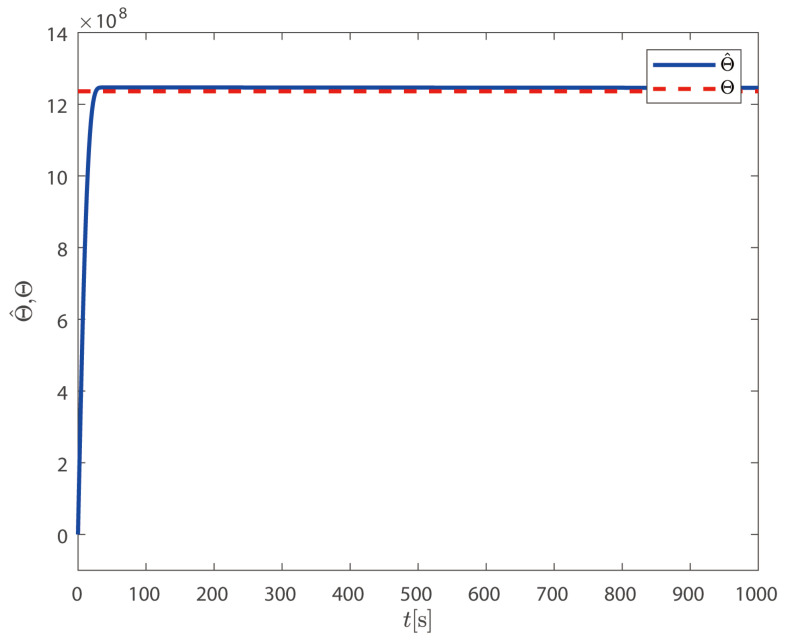
Estimated and real values of the single-parameter learning strategy.

According to Remark 1, the ship model parameters *M* and *D* are uncertain, and the disturbance *d* is unknown. On this basis, the composite uncertainties are used to contain the above uncertainties. In order to design an effective DP control law, only one unknown virtual parameter needs to be adjusted online, which solves the influence of uncertainties. The simulation results of the single parameter learning neural network are shown in Figure 14.

## 6. Discussion

The simulation results validate the effectiveness of the proposed minimal-sensor adaptive DP control strategy through three key aspects. First, the sliding mode observer successfully achieves reliable state estimation without additional sensors by effectively filtering high-frequency motion components while reconstructing low-frequency position and heading information. This significantly reduces system complexity and cost compared to traditional sensor-dependent approaches.

Second, the output feedback control demonstrates clear advantages over conventional state feedback methods in disturbance handling. Although both approaches achieve the desired positioning objectives, the output feedback control exhibits notably smaller oscillation amplitudes and reduces actuator wear through effective high-frequency motion filtering. The incorporation of thruster system dynamics further enhances control performance by generating smoother control signals compared to previous methods.

However, certain limitations exist. The output feedback control introduces slight positioning overshoot, and the system may face challenges in extremely harsh sea conditions where precise state estimation becomes more difficult. Future research should focus on enhancing system robustness for severe environmental conditions, investigating multi-vessel coordination scenarios, and integrating advanced learning algorithms to improve adaptation in dynamic marine environments.

## 7. Conclusions

This paper presents a novel adaptive dynamic positioning control strategy based on multi-observer fusion with minimal sensor requirements, with several significant contributions. Firstly, the paper develops a dual-observer fusion architecture that combines sliding mode observers with finite-time convergence disturbance observers, achieving comprehensive state estimation and disturbance rejection with reduced sensor dependency. This approach effectively enhances the ship’s stable positioning capability under complex sea conditions. Secondly, by introducing an auxiliary dynamic system, the problem of actuator input saturation is addressed, and the dynamic equation of the actuator system is established, enhancing the applicability of the control algorithm in practical engineering. Additionally, the paper implements a single-parameter learning neural network to handle model uncertainties, requiring only the online estimation of one parameter, which significantly reduces computational complexity and controller implementation costs.

Future research will focus on extending the proposed control strategy to multi-vessel coordination scenarios, investigating inter-vessel interference patterns and fleet synchronization challenges. Further development will explore the integration of advanced learning algorithms with the current observer-based framework to enhance system adaptation and reliability in dynamic marine environments.

## Figures and Tables

**Table 1:** Sample data.

	Thrusters	Limit Values
Insurge	$\tau_{M1}$	$3.76815\times 10^{2}\;\mathrm{kN}$
Insway	$\tau_{M2}$	$6.8072\times 10^{2}\;\mathrm{kN}$
Inyaw	$\tau_{M3}$	$7.31\times 10^{2}\;\mathrm{kN}$

## Data Availability

The data presented in this study are contained within the article.
